# Diverse organization of voltage-gated calcium channels at presynaptic active zones

**DOI:** 10.3389/fnsyn.2022.1023256

**Published:** 2022-12-05

**Authors:** Weijia Zhang, He-Hai Jiang, Fujun Luo

**Affiliations:** ^1^Guangzhou Laboratory, Guangzhou, China; ^2^Division of Life Sciences and Medicine, University of Science and Technology of China, Hefei, China; ^3^Bioland Laboratory, Guangzhou, China

**Keywords:** calcium channels, nanodomain, microdomain, neurotransmitter release, synaptic computation, synaptic plasticity, modulation, active zone

## Abstract

Synapses are highly organized but are also highly diverse in their organization and properties to allow for optimizing the computing power of brain circuits. Along these lines, voltage-gated calcium (CaV) channels at the presynaptic active zone are heterogeneously organized, which creates a variety of calcium dynamics profiles that can shape neurotransmitter release properties of individual synapses. Extensive studies have revealed striking diversity in the subtype, number, and distribution of CaV channels, as well as the nanoscale topographic relationships to docked synaptic vesicles. Further, multi-protein complexes including RIMs, RIM-binding proteins, CAST/ELKS, and neurexins are required for coordinating the diverse organization of CaV channels at the presynaptic active zone. In this review, we highlight major advances in the studies of the functional organization of presynaptic CaV channels and discuss their physiological implications for synaptic transmission and short-term plasticity.

## Introduction

Synapses are the structural and functional units that compute and transfer information between individual neurons in brain circuits. For fast synaptic transmission, voltage-gated calcium (CaV) channels are activated by membrane depolarization to conduct calcium influx into the nerve terminal, where a highly complex series of biophysical and biochemical events take place within milliseconds to release neurotransmitters (for review, see Südhof, 2004). CaV channels are tightly assembled within a spatially restricted, highly specialized region of the terminal, called the active zone (for review, see Südhof, 2012; Emperador-Melero and Kaeser, 2020), to achieve the high speed and precision of synaptic transmission. Compelling evidence has revealed that CaV channel subtype, number, and distribution as well as their nanoscale topographic relationship to synaptic vesicles (SV) in presynaptic active zones are heterogeneously regulated. The heterogeneity produces a huge diversity of calcium dynamics at the release sites in different synapses, and thus endows individual synapses with different functions that are essential for synaptic computation within neural circuits. This review focuses on recent progress in our understanding of the organization of CaV channels at the presynaptic active zone, their underlying molecular mechanisms, as well as the functional implications for synaptic transmission and short-term plasticity.

## CaV Channels at The Presynaptic Active Zone

**Figure 1:**
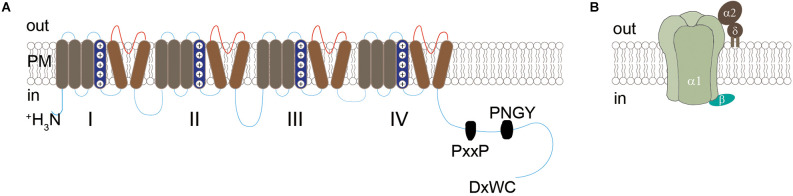
Diagrammatic structure of CaV channels. **(A)** Structure of the α1 subunit, which contains four homologous domains (I-IV) each with six transmembrane segments (S1-S6) and a long intracellular C-terminal. The cytoplasmic tail contains the conserved PxxP motif, PNGY motif, and C-terminal motif DxWC, which are known to be crucial for the synaptic assembly of CaV2 channels (see details in text). **(B)** Model of CaV channels showing the central pore-forming α1 subunit, interacting with intracellular β subunit and extracellular α_2_δ subunit through a GPI anchor into the plasma membrane.

CaV channels are transmembrane proteins that couple membrane depolarization with calcium influx to trigger various cellular activities including transmitter release. They are composed of a single pore-forming primary α_1_ subunit, an intracellular β subunit, and an extracellular α_2_δ subunit (Figure 1). The α_1_ subunits are encoded by 10 genes, and the resulting CaV channels can be classified into three subfamilies: CaV1, CaV2, and CaV3. The CaV1 subfamily includes CaV1.1, CaV1.2, CaV1.3, and CaV1.4, which conduct L-type calcium currents. The CaV2 subfamily includes CaV2.1, CaV2.2, and CaV2.3, which conduct P/Q-type, N-type, and R-type calcium currents, respectively. The CaV3 family includes CaV3.1, CaV3.2, and CaV3.3, which conduct T-type calcium currents. The diversity of CaV channels is further increased by the expression of four genes that code for β subunits, four genes that code for α_2_δ subunits, and their alternative splice variants (for review, see Catterall and Few, 2008).

Fast transmitter release is almost exclusively mediated by calcium influx through CaV2 channels at mammalian synapses, with the exception that auditory and retina ribbon synapses are dominated by CaV1 (Brandt et al., 2003; for review, see Moser et al., 2020). All three Ca2 channels including CaV2.1, CaV2.2, and CaV2.3 may coexist and contribute to transmitter release at the same synapse (Takahashi and Momiyama, 1993; Wheeler et al., 1994; Mintz et al., 1995; Wu et al., 1998; Iwasaki et al., 2000; Pelkey et al., 2006; Li et al., 2007; Rebola et al., 2019). Direct recording of presynaptic calcium currents has been achieved in hippocampal mossy fiber boutons (MFBs) and the calyx of Held synapse in the auditory brain stem, and these studies have enabled a comparison of the functional properties of different CaV2 subtypes in the native environment (Li et al., 2007; Sheng et al., 2012). The results have generally suggested that all CaV2 channels share similar biophysical properties including activation/deactivation kinetics and opening probability (Sheng et al., 2012; but see Li et al., 2007). However, these studies have primarily focused on CaV2.1 and CaV2.2 channels, while CaV2.3 channels appear to contribute very little (~10%) to basal calcium current and transmitter release in most if not all synapses and remain less understood and will not be further discussed (Dietrich et al., 2003).

Although CaV2.1 and CaV2.2 channels mediate the majority of neurotransmitter release across different synapses and developmental stages, accumulating studies have revealed that the contribution of each type in controlling transmitter release at individual synapses may vary dramatically (Wu et al., 1998; Iwasaki et al., 2000; Pelkey et al., 2006; Li et al., 2007; Arai and Jonas, 2014; Lubbert et al., 2019; Rebola et al., 2019; Held et al., 2020). Some synapses utilize both CaV2.1 and CaV2.2 channels additively to mediate transmitter release. For example, at the hippocampal mossy fiber synapses on both CA3 pyramidal cells and interneurons, CaV2.1 channels primarily mediate neurotransmitter release (~80%) with a minor but significant contribution of CaV2.2 channels (~20%; Wu et al., 1998; Pelkey et al., 2006). At the Schaffer collateral synapses, however, both CaV2.1 and CaV2.2 equally mediate neurotransmitter release (Castillo et al., 1994; Wu and Saggau, 1994). On the other extreme, CaV2.1 or CaV2.2 can be exclusively expressed in individual synapses that are typically associated with distinct release properties. For example, CaV2.1 channels exclusively mediate synchronous GABA release in parvalbumin (PV)-interneurons whereas CaV2.2 channels are fully responsible for not only synchronous but also asynchronous GABA release in cholecystokinin (CCK)-interneurons (Hefft and Jonas, 2005). Similarly, it has been found in zebrafish that CaV2.1 and CaV2.2 specifically mediate neurotransmitter release in two functionally distinct motoneurons which postsynaptically innervate the same muscle fiber (Wen et al., 2020).

## The Number of Presynaptic CaV Channels

Accumulating studies have revealed that not only the subtype of CaV2 channels but also the number or density of CaV channels expressed at the presynaptic active zone is a critical determinant of synaptic function (Holderith et al., 2012; Sheng et al., 2012). It is worth noting that it remains a significant challenge to measure accurately the number of presynaptic CaV channels. Estimates of the abundance of CaV channels within each active zone typically rely on combined experimental and computational approaches in model synapses which are large and relatively accessible. For instance, at the frog neuromuscular synapse, calcium imaging and optical fluctuation analysis have estimated that an average of 36 CaV channels function within each active zone (Luo et al., 2011). Interestingly, at the young calyx of Held synapse, cell-attached patch clamp recording of calcium currents of single active zones found a wide range in the number of CaV channels (from 5 to 218 per active zone; Sheng et al., 2012). Remarkably, an elegant study utilizing two-photon calcium imaging with optical fluctuation analysis has recently measured the number of CaV channels in cortical synapses, which typically have much smaller terminals (on the order of 1 μm in diameter) and contain only a single active zone (Rebola et al., 2019). In this study, an average of ~236 CaV channels were estimated at individual boutons of cerebellar granule cells (GCs) whereas an average of only 65 CaV channels were estimated at individual boutons of cerebellar stellate cells (SCs; Rebola et al., 2019), suggesting a similar scenario such that there is a large variability of the number of CaV channels in cortical synapses.

Since CaV channels directly mediate calcium influx for triggering transmitter release, the abundance of CaV channels in individual active zones has a significant impact on the functional properties of synaptic release probability and plasticity. Indeed, the number of CaV channels has been shown to correlate linearly with transmitter release strength by controlling not only the release probability of individual readily-releasable vesicles but also the total number of readily-releasable vesicles (Sheng et al., 2012). Two-photon calcium imaging and correlated electron microscopic ultrastructure reconstruction of rat hippocampal synapses further revealed a positive correlation of both the peak calcium transient in single boutons and the release probability with the active zone area (Holderith et al., 2012). The number of immunogold labeled the CaV2.1 channels scales linearly with the size of the active zone (Holderith et al., 2012). Based on these studies, it seems reasonable to predict that a larger active zone should have a higher number of CaV channels, a higher number of readily-releasable vesicles, and thus a higher release probability in most synapses.

Counterintuitively, at some synapses, the opposite may hold true as revealed recently by comparative studies of cerebellar GC and SC synapses. The GC synapses are weaker in release probability even though they have a stronger AP-evoked calcium influx due to the presence of a higher number of CaV channels as compared to the SC synapses (Rebola et al., 2019). Such a discrepancy appears to correlate with the tighter coupling of CaV channels and synaptic vesicles in the SC synapses than in the GC synapses (see below for further discussion).

Regardless of extensive studies of CaV2 subtypes mediating transmitter releases at various synapses, the molecular mechanisms for selective targeting of particular subtypes of CaV channels at individual synapses remain largely unknown. One prevailing view is that different CaV channel “slots” are present in different synapses (Cao et al., 2004). The slot numbers and types may set the levels and preferences respectively at synapses for CaV2 channels (Cao et al., 2004; Cao and Tsien, 2010). However, this hypothesis has recently been challenged by an elegant study that takes advantage of the calyx of Held synapse in combination with virus-based overexpression of CaV2.1 and CaV2.2 channels. Comparative analysis of functional presynaptic calcium currents and freeze-fractured immunogold labeling of CaV2.1 α_1_ subunits demonstrated that CaV2.1 α_1_ subunit overexpression could increase synaptic calcium current, CaV2.1 channel number, and thus synaptic strength at both developing and mature synapses, arguing against a CaV2 channel slot preference and saturation hypothesis (Lubbert et al., 2019).

## Topographic Organization of CaV Channels

Neurotransmitter release at central synapses is highly sensitive to action potential-evoked calcium influx and the release rate increases superlinearly (3–4th power relationship) with the rise of calcium (Dodge and Rahamimoff, 1967; Borst and Sakmann, 1996; Bollmann et al., 2000; Schneggenburger and Neher, 2000). Additionally, action potential-evoked calcium rise at release sites is extremely brief and localized (Sabatini and Regehr, 1996), primarily because CaV channels are not randomly distributed but clustered within the presynaptic active zone (Holderith et al., 2012; Nakamura et al., 2015). Therefore, the spatial relationship between CaV channel clusters and synaptic vesicles is crucial for shaping the release properties of synapses.

Mounting evidence has shown that the CaV-SV topography is also subject to specific control and varies significantly across different synapses. Broadly, distinct CaV-SV topographies can be differentiated into “microdomain” or “nanodomain” configurations (for review, see Neher, 1998; Eggermann et al., 2011). Experimental works on a large variety of synapses have examined the coupling tightness between CaV channels and SVs by comparing the blocking effectiveness of high concentrations of calcium chelators on transmitter release (Adler et al., 1991; Hefft and Jonas, 2005; Bucurenciu et al., 2008; Eggermann et al., 2011; Scimemi and Diamond, 2012; Schmidt et al., 2013; Arai and Jonas, 2014). BAPTA and EGTA are high-affinity calcium chelators with similar K_d_ (in the range of 70–220 nM) but display remarkably different binding kinetics (BAPTA is 40-fold faster than EGTA). EGTA even at a high concentration of 10–30 mM is not capable of chelating calcium and blocking transmitter release if the diffusion distance between open channels and calcium sensors is short. The inhibitory effect of EGTA on neurotransmission may increase as the coupling distance increases. In a majority of synapses, it has been found that only BAPTA, but not EGTA, may significantly reduce transmitter release, suggesting that CaV channels are tightly coupled with synaptic vesicles (Adler et al., 1991; Hefft and Jonas, 2005; Bucurenciu et al., 2008; Eggermann et al., 2011; Scimemi and Diamond, 2012; Schmidt et al., 2013; Arai and Jonas, 2014; Nakamura et al., 2015; Rebola et al., 2019). Nevertheless, a high concentration of EGTA can block transmitter release at a few other synapses, for example, the synapse between CCK interneurons and granule cells in the hippocampal dentate gyrus (Hefft and Jonas, 2005), the synapse between hippocampal mossy fiber boutons and CA3 pyramidal neurons (Vyleta and Jonas, 2014), the synapse between parallel fibers and Purkinje cells (Rebola et al., 2019), and the young calyx of Held synapse (Fedchyshyn and Wang, 2005). A loose microdomain coupling of CaV-SVs is proposed to explain the effectiveness of EGTA in these preparations.

Apparently, the nanodomain CaV-SV topography is important to increase the efficacy, timing, and precision of synaptic transmission. For example, selective ablation of Rim-binding proteins (RBPs) impairs the tight coupling of CaV-SV and therefore reduces the precision of timing of AP-evoked transmitter release (Acuna et al., 2015; Grauel et al., 2016). The calyx of Held synapse is well known for undergoing a developmental switch from microdomain to nanodomain CaV-SV topography (Fedchyshyn and Wang, 2005; Nakamura et al., 2015), which is thought to be critical for high-fidelity synaptic transmission of auditory processing in adult animals. Similarly, the tightening of CaV-SV coupling has been reported during the maturation of the cerebellar cortical synapse (Baur et al., 2015; but see Rebola et al., 2019). Interestingly, such developmental tightening of CaV-SV topography has also been found in Drosophila (Böhme et al., 2016), suggesting an evolutionarily conserved mechanism.

However, the “loose” configuration appears not restricted to the early developmental stage of synapses. In the P19–22 rat hippocampus, GABA release from CCK interneurons is exclusively mediated by CaV2.2 channels and dramatically depressed by EGTA (Hefft and Jonas, 2005). By contrast, GABA release from PV interneurons is mediated exclusively by CaV2.1 channels and not affected by EGTA (Hefft and Jonas, 2005). In P20–23 rats, the mossy fiber synapse with CA3 pyramidal neurons, which co-expresses both CaV2.1 and CaV2.2 channels, has been shown to be highly sensitive to EGTA blockade of transmitter release, suggesting a loose CaV-SV coupling (Vyleta and Jonas, 2014). Interestingly, this synapse is subject to robust regulation of release probability and synaptic plasticity by endogenous calcium buffers (Vyleta and Jonas, 2014). In the cerebellum of adult mice, Rebola and colleagues demonstrated that the GC-SC synapse, which co-expresses both CaV2.1 and CaV2.2 channels, displays significantly higher sensitivity to suppression by EGTA-AM, as compared to the SC-SC synapse, which expresses only CaV2.1 (Rebola et al., 2019).

Collectively, these data suggest an attractive hypothesis that at least in the mammalian brain, most synapses predominantly express CaV2.1 channels and employ tight nanodomain CaV-SV topography for transmitter release. In contrast, other synapses primarily express CaV2.2 channels, with or without co-expression of CaV2.1, and employ loose microdomain CaV-SV topography for transmitter release. There appears a strong temporal correlation between the developmental switch from loose to tight CaV-SV coupling and CaV2.1 channels becoming dominant over CaV2.2 in triggering transmitter release at the calyx of Held synapse (Iwasaki and Takahashi, 1998; Fedchyshyn and Wang, 2005), which fits well with the hypothesis. Future investigations are needed to further test this hypothesis and if so, what are the molecular candidates that may underlie distinct topography of different subtypes of CaV channels with SVs?

EM immunogold labeling of CaV channels and Munc13, which is essential for docking and priming of synaptic vesicles (Augustin et al., 1999), have been utilized to directly determine the topographical relationship between CaV channels and release sites at cerebellar synapses (Rebola et al., 2019). In the SC-SC synapse, a small number of CaV channels are clustered and distributed close to Munc13 clusters, which suggests a perimeter release model for high release probability. In contrast, in the GC-PC synapse, Munc13 clusters are not tightly surrounded by CaV channel clusters, suggesting an exclusion zone model in which CaV channels are distributed outside an area of ~50 nm of Munc13 clusters. Such different topographic organizations in CaV channels and release sites are consistent with the heterogeneous release properties observed at these two different synapses (Rebola et al., 2019). Interestingly, at the Drosophila synapses, distinct isoforms of (M)Unc13 may be recruited and clustered by separate scaffold proteins Bruchpilot (BRP) and Syd1 (Böhme et al., 2016; Fulterer et al., 2018). Experiments using two-color STED microscopy have further demonstrated that the BRP-(M)Unc13A clusters are distributed much closer to CaV channels (≈70 nm) than the Syd1-(M)Unc13B clusters (≈120 nm). Such tight- and loose coupling configurations in different synapses (Fulterer et al., 2018) or release sites (Böhme et al., 2016) are correlated well with their distinct functional properties and synaptic plasticity. Clearly, Munc13 represents a core component of release machinery and its cluster number is correlated with the number of quantal release sites (Sakamoto et al., 2018), but there seems a large variability in the density and dimension of Munc13 clusters (Karlocai et al., 2021). Therefore, it is of interest and importance to understand the quantitative molecular nature of release sites and their functional heterogeneity in the future.

## Nanoscale Modulation of CaV Channels by G-Protein Coupled Receptors

Presynaptic CaV channels are subject to a large variety of modulatory effects mediated by G-protein coupled receptors (GPCRs) for many different neurotransmitters including glutamate, GABA, endocannabinoid, and dopamine, which may exert differential and specific modulation of CaV channels and thus synaptic functions (for review, see Lovinger et al., 2022). Interestingly, multiple lines of evidence have suggested that the tightness in the coupling of GPCRs with CaV channels at presynaptic active zones may play an important role in determining the strength of GPCR signaling. For example, EM studies of immunogold labeling have shown that metabotropic glutamate receptors (mGluR4, mGluR7, and mGluR8) are localized in presynaptic active zones (Shigemoto et al., 1996; Ferraguti et al., 2005). Two-color super-resolution imaging further reveals that mGluR4 forms nanoclusters within active zones of cerebellar PFs and co-localizes with CaV2.1 channels and Munc18 (Siddig et al., 2020). Similarly, presynaptic GABA_B_-receptors are clustered within active zones and play a critical role in regulating CaV channel function (Lujan et al., 2004, 2018; Luo et al., 2021). By contrast, EM and super-resolution imaging show that CB1 receptors are excluded from active zones, but distributed uniformly in the extrasynaptic region of the nerve terminal (Nyiri et al., 2005; Dudok et al., 2015).

Elegant studies have shown that when high-frequency stimulation triggers a large glutamate release that activated presynaptic mGluR7, this in turn produces sustained inhibition of CaV2.1 channels and long-term depression of hippocampal mossy fiber synapse on interneurons (Pelkey et al., 2006). Therefore, various GPCR signaling molecules may modulate distinctively CaV channel activities and thus regulate synaptic function in synapse-specific and pathway-specific manners, increasing the complexity and richness of synaptic plasticity and circuit computation. For example, it has been demonstrated that presynaptic regulation of CaV channels by GABA_B_ receptors imposes a high-pass filter on synaptic transmission and induces facilitation at high-frequency neuronal activities whereas regulation of CaV channels by dopamine receptors suppresses synaptic transmission independent of frequency and thus specifically changes synaptic gain (Burke et al., 2018).

## Molecular Mechanisms of CaV Channel Assembly at Active Zones

**Figure 2:**
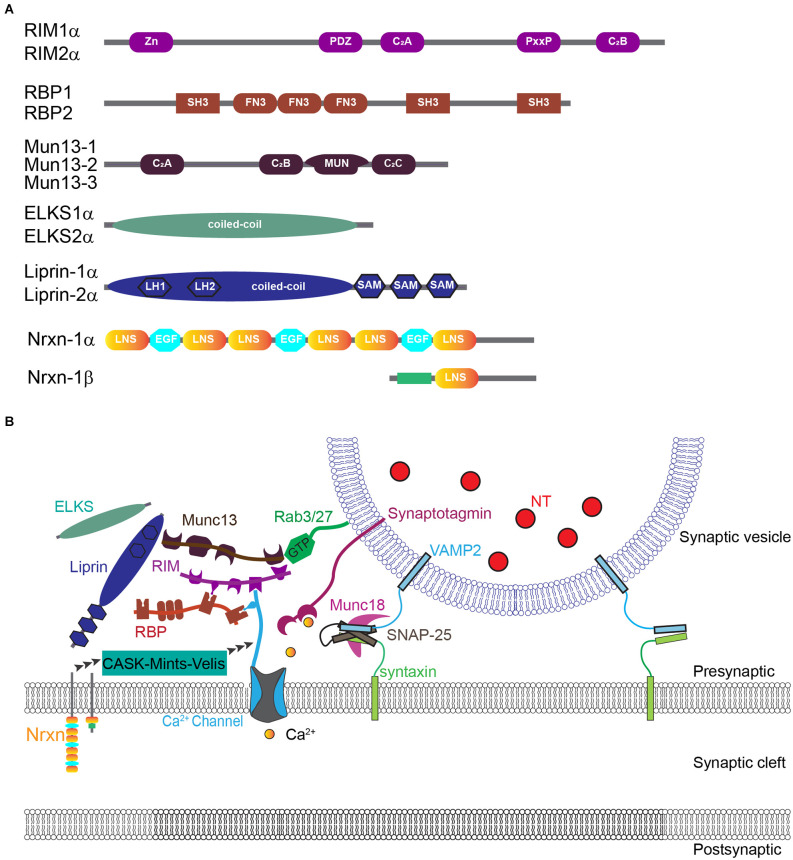
Molecular organization of CaV2 channels at the presynaptic active zone. **(A)** Diagram of domain structures of evolutionarily conserved key active zone proteins. **(B)** Model of nanoscale organization of CaV channels interacting with complex active zone proteins and synaptic vesicles. Adapted with permission from Südhof (2012).

The selective targeting and organization of CaV channels at the presynaptic active zone have been extensively studied and tremendous progress has been made in understanding the underlying molecular mechanisms (Figure 2; for excellent reviews see Südhof, 2012; Dolphin and Lee, 2020). Surprisingly, recent work from Kaeser and colleagues provides strong evidence that CaV channels are not required for synapse formation and the assembly of the presynaptic active zone (Held et al., 2020).

Strong evidence has demonstrated that the C-terminal of CaV2.1 and CaV2.2 contains multiple conserved motifs that are essential for presynaptic targeting by interacting directly or indirectly with complex protein machinery. Original studies have shown that in immature neurons, the long C-terminal splice variant of CaV2.2 is distributed diffusely and uniformly, whereas the short C-terminal variant is localized in the somatodendritic domain. After synaptogenesis, the long splice variant is clustered at synapses probably *via* interaction with Mint1 and CASK (Maximov et al., 1999; Maximov and Bezprozvanny, 2002). Similar roles of the C-terminal have been demonstrated for CaV2.1 channels (Held et al., 2020). Deletion of the entire C-terminal of CaV2.1 does not interfere with the neuronal expression of CaV2.1 channels but eliminates their synaptic localization (Held et al., 2020).

Multiple evolutionarily conserved active zone-specific proteins, including Munc13s, RIMs, RIM-BPs, ELKS, and α-liprins, are shown to directly or indirectly interact with the C-terminal of CaV2 channels to coordinate their synaptic targeting and nanoscale organization at the presynaptic active zone. For example, RIMs and RBPs play key roles in CaV channel anchoring at active zones by forming a tripartite complex with CaV channels (Han et al., 2011; Kaeser et al., 2011; Acuna et al., 2016; Oh et al., 2021). RIMs directly and selectively interact with the C-terminal PDZ binding domain of CaV2.1 and CaV2.2 channels (but not CaV1 channels) through their central PDZ domains (Kaeser et al., 2011). Deletion of all RIM1/2 isoforms strongly reduces calcium currents at the nerve terminal and thus neurotransmitter release (Han et al., 2011; Kaeser et al., 2011). RIMs can also modulate CaV channels indirectly through the interaction of the central proline-rich sequences with the SH3 domain of RBPs, which interact directly with all presynaptic CaV channel subtypes (Kaeser et al., 2011). RBP1 and RBP2 proteins interact *via* their SH3 domains not only with RIMs but also with the cytoplasmic proline-rich sequences of CaV1s and CaV2s (Hibino et al., 2002; Kaeser et al., 2011; Muller et al., 2015). In Drosophila, deletion of RBP reduces CaV channel abundance and calcium influx, decreases the number of docked vesicles, disrupts the integrity of the active zone cytomatrix, and dramatically impairs calcium-triggered neurotransmitter release (Liu et al., 2011; Muller et al., 2015). In contrast, deletion of RBP1 and RBP2 in mice does not influence the intrinsic properties, or the density of presynaptic CaV2.1 and CaV2.2 channels, and produces only mild impairments in the extent of transmitter release in response to single spikes (Acuna et al., 2015; Grauel et al., 2016). Remarkably, however, the deletion of RBPs from murine synapses strongly and specifically disrupts the coupling of CaV channels to synaptic vesicle exocytosis and renders action potential-mediated transmitter release highly unreliably during spike trains (Acuna et al., 2015; Grauel et al., 2016). Deletion of ELKS1/2 is shown to impair normal Ca influx for neurotransmitter release without changing CaV channels tethering in presynaptic active zones (Liu et al., 2014).

Neurexins are key presynaptic cell adhesion molecules essential for synapse formation and function through interaction with many distinct postsynaptic ligands (Missler et al., 2003; Südhof, 2008; Chen et al., 2017). Neurexins can bind with MINT and CASK (Hata et al., 1996; Butz et al., 1998; Biederer and Südhof, 2000), two scaffold proteins interacting directly with CaV channels (Maximov et al., 1999). Triple knockout of α-neurexins causes a significant reduction of calcium current especially mediated by CaV2.2 (Missler et al., 2003; Zhang et al., 2005), suggesting that neurexins play an important role in the regulation of presynaptic CaV channels. Interestingly, in the prefrontal cortex, pan-neurexin deletion causes a remarkable decrease in action potential-evoked calcium transients in presynaptic boutons of somatostatin interneurons, but not in PV interneurons (Chen et al., 2017). Recent work using patch-clamp recording at the calyx of Held synapse did not reveal a significant change in presynaptic calcium currents, but instead found a prominent change in CaV-SV coupling after removing all three α- and β-neurexins (Luo et al., 2020). These results suggest that neurexins may play diverse roles in modulating presynaptic CaV channels and thus synaptic functions depending on the specific expression of various pre- and postsynaptic molecules in different synapses.

Similarly, the active zone scaffold protein Bassoon has been shown to interact directly with RBPs, but not with CaV2.1 or CaV2.2 channels. Interestingly, such interaction of Bassoon and RBPs may specifically cluster CaV2.1 channels to the presynaptic active zone of hippocampal neurons, without a direct impact on CaV2.2 (Davydova et al., 2014). However, Bassoon’s role in the specific recruitment of CaV2.1 channels needs to be verified and the underlying molecular mechanism remains to be clarified since Bassoon is widely expressed in most synapses, and RBPs bind strongly with both CaV2.1 and CaV2.2 channels.

Altogether, the proper expression and organization of CaV channels are subject to coordination and redundant control of many specific molecules (Kittel et al., 2006; Han et al., 2011; Kaeser et al., 2011; Liu et al., 2011; Davydova et al., 2014; Acuna et al., 2016; Wang et al., 2016; Luo et al., 2020). Ablation of these key molecules leads to strong impairment of synaptic transmission, which can be caused by either reduced levels or mislocalization of CaV channels in the presynaptic active zone. It is worth pointing out, however, that the tethering of CaV channels to active zones seems quite robust such that even simultaneous interference of CaV channel interaction with multiple key proteins may not completely block presynaptic calcium currents (Acuna et al., 2016; Wang et al., 2016; Lubbert et al., 2017), suggesting parallel and redundant mechanisms implemented in CaV channels targeting to active zones (Held et al., 2020).

## Conclusions

Advances in multi-disciplinary techniques such as freeze-fracture immunogold electron microscopy, super-resolution imaging, two-photon calcium imaging, and patch-clamp recording have greatly enhanced our understanding of the nanoscale organization and function of presynaptic CaV channels in synaptic transmission and plasticity. Substantial research has shed light on how CaV channels are specifically assembled to achieve dynamic control of transmitter release across different synapses. One emerging principle is that presynaptic CaV channels are subject to tight control by exquisite molecular machinery during development and in different synapses. The heterogeneity in the subtype, number, distribution, and topographic relationship of CaV channels with synaptic vesicles generates a wide spectrum of calcium signals in different synapses that can tune their release strength, kinetics, timing, and plasticity.

Although we have gained a great deal of knowledge of how multiple key molecules can coordinate to form a core complex that assembles CaV channels properly within the presynaptic active zone, the list of molecules that are involved in forming this complex is not complete. Further, individual components of such a complex may vary significantly across different synapses and thus account for the large variability in the properties and functions of different synapses in brain circuits. These issues need to be addressed in future studies.

## Author Contributions

WZ, H-HJ, and FL wrote the article. FL made the figures and edited the manuscript before submission. All authors contributed to the article and approved the submitted version.

## Funding

This work was supported by the National Natural Science Foundation of China (31970914 to FL).

## Conflict of Interest

The authors declare that the research was conducted in the absence of any commercial or financial relationships that could be construed as a potential conflict of interest.

## Publisher’s Note

All claims expressed in this article are solely those of the authors and do not necessarily represent those of their affiliated organizations, or those of the publisher, the editors and the reviewers. Any product that may be evaluated in this article, or claim that may be made by its manufacturer, is not guaranteed or endorsed by the publisher.
